# Long-Term Aerobic Exercise Protects against Cisplatin-Induced Nephrotoxicity by Modulating the Expression of IL-6 and HO-1

**DOI:** 10.1371/journal.pone.0108543

**Published:** 2014-10-01

**Authors:** Mariana Yasue Saito Miyagi, Marilia Seelaender, Angela Castoldi, Danilo Candido de Almeida, Aline Villa Nova Bacurau, Vinicius Andrade-Oliveira, Lucas Maceratesi Enjiu, Marcus Pisciottano, Caroline Yuri Hayashida, Meire Ioshie Hiyane, Patricia Chakur Brum, Niels Olsen Saraiva Camara, Mariane Tami Amano

**Affiliations:** 1 Laboratory of Immunobiology of Transplants, Department of Immunology, Institute of Biomedical Sciences, University of Sao Paulo, Sao Paulo, SP, Brazil; 2 Cancer Metabolism Research Group, Department of Cell Biology, Institute of Biomedical Sciences, University of Sao Paulo, Sao Paulo, SP, Brazil; 3 Laboratory of Molecular and Cellular Exercise Physiology, School of Physical Education and Sport, University of Sao Paulo, Sao Paulo, SP, Brazil; Institut national de la santé et de la recherche médicale (INSERM), France

## Abstract

Nephrotoxicity is substantial side effect for 30% of patients undergoing cancer therapy with cisplatin and may force them to change or even abandon the treatment. Studies regarding aerobic exercise have shown its efficacy for the treatment of many types of diseases and its capacity to reduce tumors. However, little is known about the impact of physical exercise on cisplatin-induced acute kidney injury (AKI). In the present study, our aim was to investigate the role of physical exercise in AKI induced by cisplatin. We submitted C57Bl6 male mice to seven weeks of chronic exercise on a training treadmill and treated them with single i.p. injection of cisplatin (20 mg/kg) in the last week. Exercise efficacy was confirmed by an increased capillary-to-fiber ratio in the gastrocnemius muscle of exercised groups (EX and CIS-EX). The group submitted to exercise before cisplatin administration (CIS-EX) exhibited less weight loss and decreased serum urea levels compared to the cisplatin group (CIS). Exercise also showed a protective role against cisplatin-induced cell death in the kidney. The CIS-EX group showed a lower inflammatory response, with less TNF and IL-10 expression in the kidney and serum. In the same group, we observed an increase of IL-6 and HO-1 expression in the kidney. Taken together, our results indicate that chronic aerobic exercise is able to attenuate AKI by inducing IL-6 and HO-1 production, which results in lower inflammatory and apoptotic profiles in the kidney.

## Introduction

Cisplatin is a potent chemotherapy drug widely used for the treatment of many types of cancers, including ovarian, head and neck cancers, and is especially effective for testicular cancer, with a cure rate greater than 90% [1], [2]. It is believed that cisplatin crosses through the plasma membrane, mostly through passive diffusion, and that once the chlorine atoms are hydrolyzed, the molecule becomes very reactive and binds to the DNA, blocking the replication of cancer cells and leading to cell death [1]. Nevertheless, cisplatin use is limited by cisplatin-acquired resistance and side effects such as acute kidney injury (AKI) [1], [3] and cachexia [4]. The latter is a catabolic syndrome that results in the involuntary weight loss involving the loss of muscle mass and of adipose tissue [5].

Unbound cisplatin molecules are filtered and actively transported, primarily by proximal tubular epithelial cells [6]. The mechanisms of its nephrotoxic remain unclear, but it is known that it involves reactive oxygen species (ROS)-mediated oxidative stress, extrinsic and intrinsic apoptosis pathways, inflammation and fibrogenesis. These factors lead to tubular damage, sodium and potassium dysfunction, and magnesium wasting [6], [7]. The inflammation process is orchestrated by tumor necrosis factor alpha (TNF), which binds to the TNFR1 and TNFR2 receptors and leads to the recruitment of two complexes. Complex 1 activates the transcription of nuclear factor–kB (NF-kB) and several inflammation and survival-related genes, while complex 2 promotes the activation of caspase-8 and -10, resulting in the induction of apoptosis [6]. TNF, normally undetectable in healthy kidneys, is produced by most renal cells in response to stimuli such as cisplatin [8], [9]. To restrain TNF damage, regulatory molecules are also produced under stress conditions. IL-10 has been described as an anti-inflammatory cytokine and plays an important role in AKI protection [10], [11]. Cisplatin treatment induces IL-10 and IL-10 receptor expression, and this endogenous production is essential for preventing damage to the renal tissue [10].

In response to endurance exercise, skeletal muscle releases cytokines such as IL-6, that promote the release of other anti-inflammatory cytokines such as IL-10 and IL-1 receptor antagonist (IL-1ra) and inhibit IL-1b and TNF production [12]. IL-6 has also been shown to protect against cisplatin-induced AKI [13]–[15].

The enzyme heme oxygenase (HO)-1 is a known regulator of the inflammatory response. It catalyzes heme degradation, thus releasing biliverdin, iron and carbon monoxide. HO-1 is associated with cytoprotection in several kidney diseases, including AKI induced by drugs [16]. Skeletal muscle cells are able to express HO-1 [17] and endurance training induces HO-1 expression in multiple immune cells [18]. Although physical exercise is able to induce HO-1, thus far, the correlation between exercise and the prevention of kidney diseases has not been established.

Physical exercise has been always recognized for its health benefits. Only in recent decades has a better understanding developed of the health benefits of physical exercise regarding the immune system and the alleviation of disability from various diseases, including obesity, diabetes mellitus, cardiovascular disease and cancer [19], [20]. Recently, the favorable effects of exercise on the efficacy of oncologic therapies and the role of exercise in the palliative care of patients with cancer have been investigated most intensively [19].

Because physical exercise contributes to a decreased inflammatory response and improves cachexia symptoms, we intended to verify whether physical exercise in mice could diminish cisplatin-induced AKI. This study could suggest new approaches for patients under cisplatin treatment.

## Objectives

Our aim was to investigate whether physical exercise was able to diminish cisplatin-induced AKI and to elucidate the possible mechanisms involved in this protection.

## Methods

### Aerobic exercise and cisplatin administration

C57Bl6 male mice, provided by UNIFESP-CEDEME, between 6 and 8 weeks of age were kept in individual cages in a vivarium with a light/dark cycle of 12/12 hours at 25±2°C, with food and water *ad libitum.* The experimental procedures were carried out according to the ethical principles for animal experimentation adopted by the Guide for the Care and Use of Laboratory Animals of the National Institutes of Health. Protocols were approved by The Ethics Committee of The Institute of Biomedical Sciences of the University of Sao Paulo – “Comissão de Ética no Uso de Animais” (CEUA) (number 046 over sheet 102 of the book 02, on April 20^th^, 2011). All efforts were made to minimize suffering. The aerobic exercise was performed on a training treadmill adapted for mice at the Laboratory of Cancer Metabolism, at the Institute of Biomedical Sciences of the University of Sao Paulo. All animals participated in exercise training for one week and were divided into four groups. Animals with best performances during the first week were selected to exercised groups. Cisplatin groups were picked randomly. These groups were: a sedentary control group (CT, *n* = 9), an exercised group (EX, *n* = 10), a cisplatin sedentary group (CIS, *n* = 8) and a cisplatin exercised group (CIS-EX, *n* = 11) according to their training. To evaluate the effects of chronic exercise, we adapted the protocol from Bacurau *et al.* [21], as shown in Table 1.

**Table 1:** Chronic training.

	Monday	Tuesday	Wednesday	Thursday	Friday
Speed	10 m/min	10 m/min	15 m/min	15 m/min	20 m/min
**Adaptation week**	-	-	30 min	35 min	30 min
**1^st^ week**	30 min	45 min	50 min	55 min	60 min
**2^nd^ week**	60 min	60 min	60 min	60 min	60 min
**3^rd^ week**	50 min	55 min	60 min	60 min	60 min
**4^th^ week**	60 min	60 min	60 min	60 min	60 min
**5^th^ week**	50 min	55 min	60 min	60 min	60 min
**6^th^ week**	60 min*	60 min	-	-	**harvest**

*Cisplatin administration.

### Cisplatin treatment

Acute kidney injury and cachexia are known side effects of cisplatin treatment. To analyze the effects of chronic exercise in this model, a single intraperitoneal (i.p.) dose of cisplatin (20 mg/kg – “Citoplax” Bergamo^©^) was administered. Mice were weighed and sacrificed 96 hours after drug administration by i.p. anesthesia of ketamine-xylazine and cervical dislocation. Immediately after sacrifice, blood, muscle and kidney were collected, frozen in liquid nitrogen, and stored at −80°C until subsequent measurements. Urea levels in the blood were verified using colorimetric assay (Labtest, Brazil) read with a Synegy microplate reader (BioTek, USA).

### Muscle capillarity

After deparaffinization, slides of gastrocnemius muscle were washed in water for 10 min and then oxidized with 0.5% periodic acid for 10 min. They were washed second time, treated with the Schiff reagent and incubated for 15 min. A third wash was performed, and the sections then were stained with hematoxylin. Briefly, capillary-to-fiber ratio was quantified by a 10×10 grid optically superimposed on each of 5 nonoverlapping fields at x400 magnification, distributed in a random manner using a computer- assisted morphometric system (Quantimet 500; Leica, Cambridge, UK). For calculating capillary-to-fiber ratio, the total number of capillaries was divided by the total number of fibers counted in the same field. Only vessels with a diameter <10 µm were counted, which would largely comprise capillaries but might also include terminal arterioles or venules. All analyses were conducted by a single observer blinded to mouse identity.

### RNA extraction and quantitative PCR (qPCR)

The gene expressions of Bcl2, Bax, TNF, IL-6, IL-10 and HO-1 were analyzed by qPCR. RNA was isolated with TRIzol Reagent (LifeTechnologies, USA) according to the manufacturer’s protocol, and RNA concentrations were determined by NanoDrop (Thermo Scientific, USA). cDNA synthesis was performed using a SuperScript III RT (Life Technologies). qPCR was performed in triplicate on each sample, using a TaqMan PCR assay (LifeTechnologies), with a ABI Prism 7300 sequence detection system (Life Technologies). Gene expression of each molecule was normalized to HPRT.

### Cytometric Bead Array – CBA

CBA was performed with Mouse, Th1/Th2/Th17 BD Biosciences CBA Kit, following the manufacturer’s instructions. We used a BD FACSCanto II flow-cytometer (BD Biosciences, USA) for cell acquisition, and FCAP array software was utilized for the data analysis.

### Western Blot

Kidney cells were lysed in RIPA buffer, quantified, run on a 10% SDS-polyacrylamide electrophoresis gel and transferred onto a nitrocellulose membrane, which was incubated with primary rabbit anti–mouse Nrf2 (Santa Cruz Biotechnology, USA), HO-1 (Abcam, USA) or β-actin (Sigma-Aldrich, USA) antibodies overnight, followed by a secondary goat anti-rabbit antibody. For each molecule, the membrane was stripped and probed with the aforementioned antibodies. The bands were analyzed with the software GeneSnap (Syngene, USA) and Gene Tools (Syngene, USA).

### TUNEL assay

After deparaffinization and rehydration, kidney sections were incubated with 20 µg/mL proteinase K (Life Technologies), and washed in PBS. They were incubated with labeling solution for 1 h at 37°C. After washing, the sections were covered with 2 drops of slow fade. Image capture was performed with a Nikon ECLIPSE Ti microscope, and the analysis was performed with NS-Elements AR software.

### Immunohistochemistry

Tissue section slides were deparaffinized and were incubated in a 95°C water bath for 30 min in a 0.01 M citrate solution. After drying, we added H_2_O_2_ for 15 min and the slides were washed twice with Tris Buffered Saline with Tween 20 (TBS-T). After blocking with non-fat dry milk 5% for 30 min, anti-HO-1 (Abcam) 1∶1200 was added, and they were incubated overnight at 4°C. We washed the slides and then incubated them with secondary antibody conjugated with peroxidase (Evision-DAKO) for 30 min. They were then treated with 3-3′ diaminobenzidine tetrahydrochloride (DAB) 1∶100 (DAKO) for 40 s. Image capture was performed with a Nikon ECLIPSE Ti microscope, and the analysis was performed with NS-Elements AR software.

### Statistical analysis

One-way analysis of variance (ANOVA) with the Bonferroni post-test was used to analyze the statistical significance of the results with GraphPad Prism 5 software.

## Results

### Chronic exercise protected against renal dysfunction and cachexia

**Figure 1:**
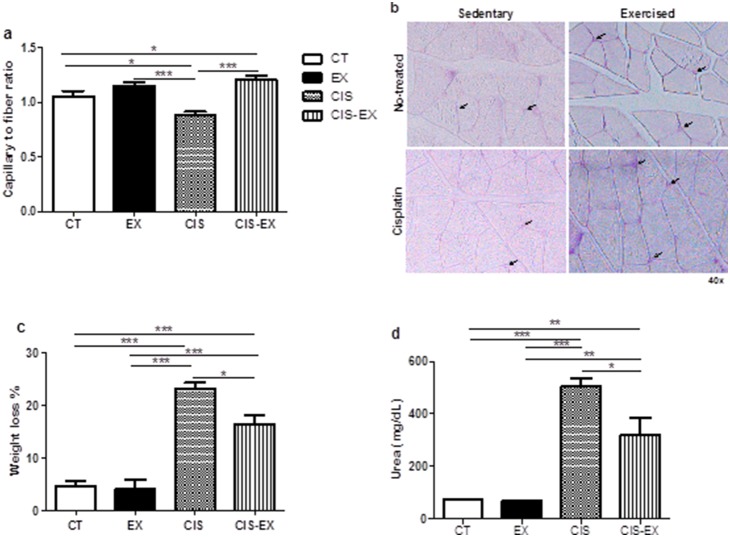
Aerobic exercise minimizes cisplatin-induced cachexia and renal damage. Exercise efficacy was confirmed by an increase in muscle angiogenesis in the exercised (EX) and exercised with cisplatin injection (CIS-EX) groups (**A** and **B**). Decreased weight loss (**C**) and lower levels of serum urea (**D**) in the CIS-EX group compared to the sedentary goup injected with cisplatin (CIS) indicate the protective effect of exercise. The sedentary group without cisplatin injection was used as the control (CT). *P<0.05; **P<0.01; ***P<0.001.

Because chronic exercise training causes physiological and metabolic stress, it is a strong proangiogenic stimulus in skeletal muscle [22]. We therefore evaluated the exercise efficiency by muscle capillary growth in gastrocnemius muscle (Figure 1A and B), and we observed an increase of capillary-muscle fiber ratio in animals submitted to exercise (EX and CIS-EX, *n* = 10 and 11, respectively). We then analyzed the influence of exercise training against the side effects of cisplatin, such as cachexia and nephrotoxicity. As depicted in Figure 1C, we observed that aerobic exercise could attenuate cisplatin-induced body weight loss, the most prominent symptom of cachexia. CIS-EX showed a lower percentage of weight loss compared to CIS (*n* = 8). After 96 h of cisplatin injection, blood was collected to evaluate renal function by determining the serum urea levels. As shown in Figure 1C, the serum urea levels increased approximately seven-fold in cisplatin-treated animals (CIS), while exercise attenuated renal dysfunction, decreasing urea levels 1.6-fold compared to CIS group. Groups not treated with cisplatin (CT and EX, *n* = 9 and 10, respectively) did not show cachexia-related weight loss or nephrotoxicity under both the sedentary and exercised conditions (Figure 1C and D).

### Exercise reduced cell death in the kidney

**Figure 2:**
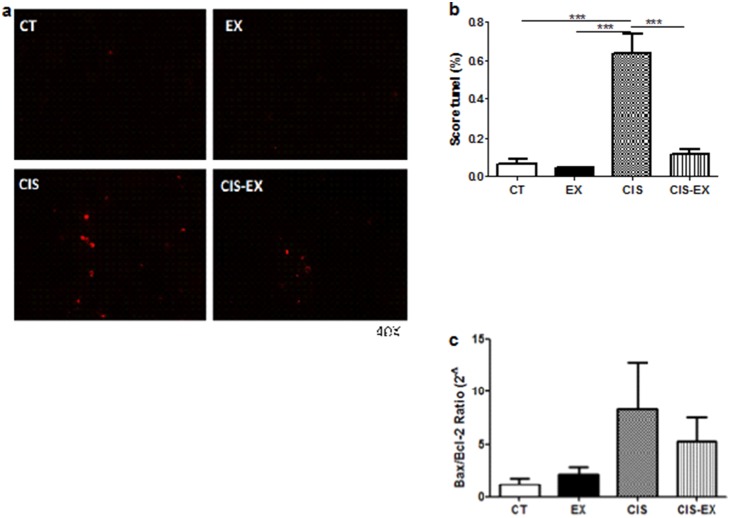
Aerobic exercise reduces cell death in the kidney. Representative TUNEL staining (**A**) and quantification (**B**) showed a significantly reduced percentage of TUNEL-positive cells (dead cells) in the CIS-EX group. The expression of Bcl2 and Bax genes was evaluated by qPCR and is represented by the ratio of the pro- to the anti-apoptotic molecule (Bax/Bcl2) (**C**) the CIS group was more likely to exhibit a pro-apoptotic profile, confirming the protective role of exercise. ***P<0.001.

To determine if aerobic exercise also had an impact on cisplatin-induced cell death, we analyzed apoptosis in the kidney. As shown in Figure 2 (A and B), cisplatin induced kidney cells apoptosis in the sedentary CIS group, while exercised mice (CIS-EX) were protected from this cell death in the kidney, as no difference was observed when compared to control groups (CT or EX). In the attempt to better understand the mechanism of this protection, we analyzed gene expression of the anti-apoptotic molecule Bcl2 and of the pro-apoptotic molecule Bax. But we found no difference among the groups (Figure 2C) by evaluating the ratios of these molecules.

### Exercise attenuated inflammation by down-modulation of TNF

**Figure 3:**
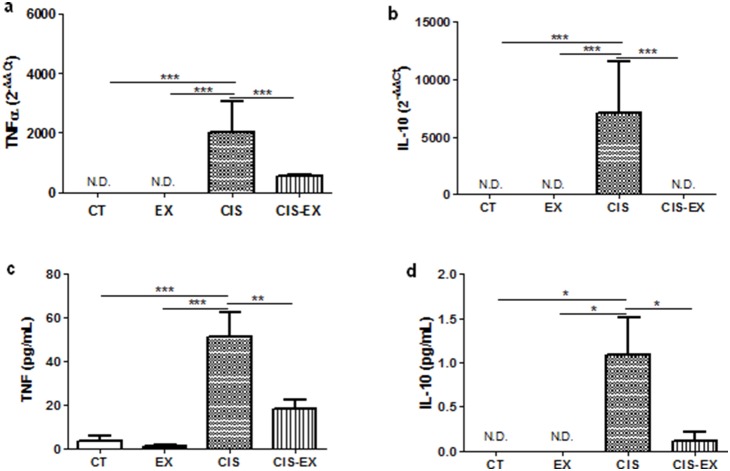
Exercise attenuates inflammation. qPCR analysis revealed that the CIS group showed higher expressions of TNF (**A**) and IL-10 (**B**) in the kidney, while CIS-EX did not exhibit significant differences compared to CT. The serum concentrations of TNF (**C**) and IL-10 (**D**), determined by CBA, appeared to decrease in the CIS-EX group. *P<0.05; **P<0.01; ***P<0.001; N.D. - non detected cytokine.

To determine that exercise was able to modulate inflammation, we analyzed the gene expression of TNF (Figure 3A) and IL-10 (Figure 3B) in the kidney as well as the levels of these two cytokines in serum (Figures 3C and D). Cisplatin increased the levels of both cytokines in the kidney and in the serum. Moreover, animals previously exposed to exercise showed decreased levels of these cytokines, indicating lower level of inflammation (Figure 3A and C). Also, a reduced need to control inflammation was observed, represented by the low levels of IL-10 (Figure 3B and D), a known regulatory cytokine.

### Exercise increased IL-6 gene expression after cisplatin administration

**Figure 4:**
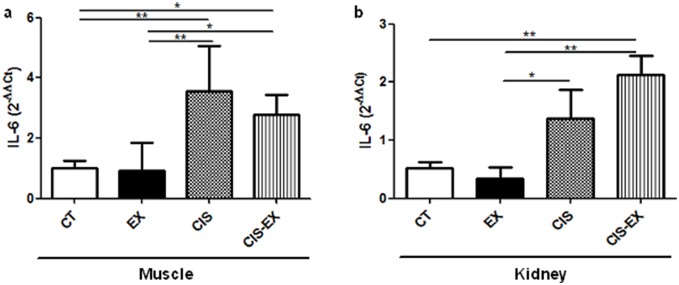
IL-6 contributes to kidney protection induced by exercise. The expression of the cytokine IL-6 was determined by qPCR in the muscle (**A**) and the kidney (**B**). The CIS group showed increased levels of IL-6 in both tissues compared to sedentary groups (CT and EX). However, CIS-EX showed increased IL-6 expression in the kidney, which indicates the protective role of IL-6 in this model. *P<0.05; **P<0.01.

IL-6 is usually associated with inflammatory response and promotes inflammation. In acute kidney injury induced by cisplatin, this cytokine seems to reduce inflammation. It was demonstrated that IL-6 plays a protective role in this model [13]–[15]. We investigated whether IL-6 plays a role in exercise-induced protection. We observed that IL-6 was expressed to a greater extent in muscle cells in the presence of cisplatin (Figure 4A), although no difference was observed between the CIS to CIS-EX groups. When we analyzed the expression of this cytokine in the kidney, we noticed that IL-6 was expressed to a greater extent in the CIS groups compared to the sedentary groups (CT and EX). However, the CIS-EX group showed even higher expression of IL-6 compared to sedentary groups (Figure 4B). This result suggests that exercised animals could result in higher levels of IL-6 in the kidney under stress conditions, promoting protection of the organ.

### Exercise increases HO-1 mRNA and protein expression in the kidney

**Figure 5:**
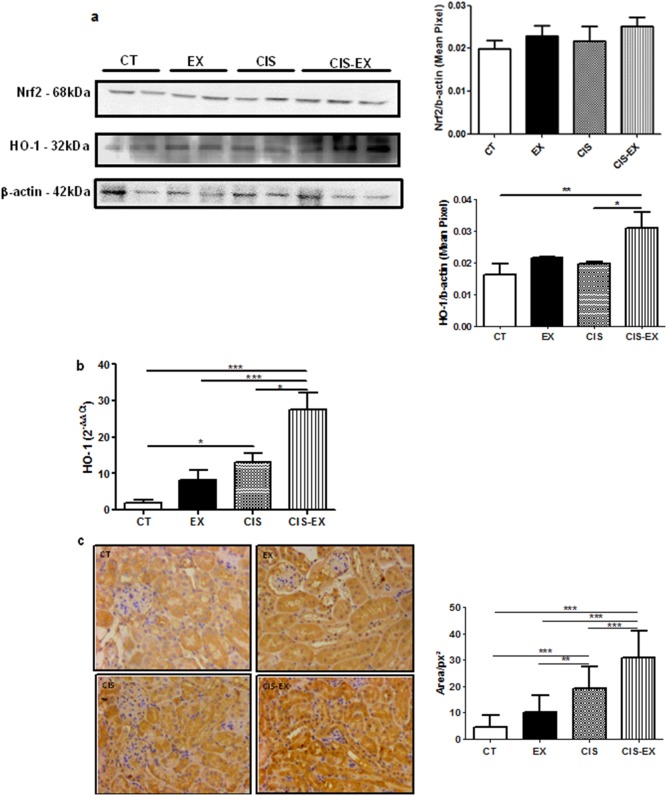
Exercise promotes increased HO-1 expression. Nrf2 and HO-1 expression in the kidney were evaluated by Western Blot (**left** – representative image and **right** – quantification). Exercise induced HO-1 gene expression, as confirmed by qPCR (**B**), and the levels of HO-1 in the renal tissue were analyzed by immunohistochemistry (**C**, **left** – representative image and **right** – quantification). *P<0.05; **P<0.01; ***P<0.001.

Because we observed less apoptosis and inflammation in the kidney of exercised animals, we questioned whether the enzyme HO-1 could be involved in the reported protective effect of chronic exercise. We first analyzed the nuclear factor erythroid-2-like 2 (Nrf2), which is a transcription factor that regulates the antioxidant response elements genes and shows effects in several inflammatory models. Nrf2 is known to induce both IL-6 [23] and HO-1 [24]. We analyzed Nrf2 production in kidney of exercised groups (Figure 5A), but no statistical significance was observed. We expected to find an increase of HO-1 in the CIS group because this enzyme is expressed during oxidative stress responses [25]. We observed increased gene expression and protein levels of HO-1 in the CIS group (Figure 5B and C). However, the exercised group (CIS-EX) showed even higher levels of HO-1 in the kidney (Figure 5A–C). Thus, we may suggest that HO-1 plays an important role in the protection induced by aerobic exercise training, by diminishing cell death and the inflammatory response in the kidney.

## Discussion

Nephrotoxicity is the primary toxic side effect of cisplatin administration, and approximately 20–30% of patients develop renal dysfunction following a single dose of cisplatin, necessitating a reduction in the dose or the treatment to be abandoned [26]. Herein, we showed that exercise in mice resulted in less chemotherapy-induced cachexia (comprising weight loss and local and systemic inflammation), a frequent consequence of cisplatin treatment, and protected the kidneys from AKI (Figure 1). These results suggest that long-term aerobic exercise might be a good strategy to improve the quality of life for chemotherapy-treated patients. Other groups have demonstrated that moderate-intensity regular exercise can efficiently attenuate fatigue in women receiving chemotherapy [17] and that resistance and aerobic exercise in patients with gastrointestinal cancer undergoing chemotherapy resulted in low scores for pain and fatigue [18]. Although studies have shown the beneficial influence of physical exercise in several diseases, the mechanisms involved are not clear, which shows the need for further study in this field.

To clarify how exercise could be contributing to kidney protection, we analyzed cell death in the renal tissue of exercised mice given cisplatin. Apoptosis in cisplatin-induced AKI occurs mainly in tubular cells as a result of ERK-dependent signalization [20]. The intrinsic pathway of apoptosis activation involves molecules such as Bax and Bad, whereas Bcl2 is an anti-apoptotic component [27]. Analyzing TUNEL staining (Figure 2A and B), we observed that CIS-EX exhibited a dramatically decreased percentage of apoptotic cells in the kidney, which contributes to decreased compromising of renal function following cisplatin treatment. The diminished cell death could be due to an increased expression of anti-apoptotic Bcl2 compared with the pro-apoptotic Bax, but in this case we found no difference on the expression of these molecules (Figure 2C), suggesting another mechanism involved.

One key protein associated with cisplatin-induced AKI is TNF, which induces apoptosis and is also a well-known pro-inflammatory cytokine [28]. TNF is produced in the kidney by many types of cells, including parenchymal cells [8], or by infiltrating cells such as T lymphocytes [29]. TNF can also be produced in response to various stimuli and binds to TNFR1 and TNFR2, thus promoting the expression of a variety of chemokines and cytokines [6]. In the present study, we observed that exercise significantly reduced TNF levels locally in the kidney as well as systemically, as observed in the serum (Figures 3A and C). We believe that this contributes to a reduction in apoptosis and a decreased inflammatory response, which also correlates with decreased weight loss due to cachexia.

IL-10 is an anti-inflammatory cytokine that shows increased levels after cisplatin injection and controls AKI progression [10], [11]. Although IL-10 is produced by adipocytes under treadmill training conditions [30] and the increased IL-10/TNF ratio indicates attenuated inflammation in cancer associated cachexia [31], no difference in IL-10 serum levels between the sedentary and exercised groups was observed. Kidneys from exercised mice did not show increased IL-10 expression (Figures 3B and D), whereas cisplatin did induce IL-10 expression in the sedentary group. Therefore, the control mechanism involved in exercised-induced protection in AKI is not associated with the IL-10 levels. We believe that the control of AKI in exercised animals occurs before the classical inflammatory response in this model, which explains the lower levels of TNF and, consequently, the decreased need for controlling the inflammation characterized by IL-10 production. These results inspired us to look for other possible mechanisms involved in this protection.

Primarily known as a pro-inflammatory cytokine, IL-6 is involved in several inflammatory disorders [32]. In acquired immunity, it plays a major role in Th17 polarization, which contributes to autoimmune diseases [33]. Although unexpected, Mitazaki *et al*. have demonstrated that IL-6 is essential for the control of cisplatin-induced injury [13]–[15]. In one study, they showed that IL-6 levels were increased in proximal tubular cells after cisplatin treatment and that IL-6 knockout (IL-6^−/−^) mice presented more severe AKI with Bax followed by an increase in Bcl-2 and Bcl-xL [15]. This same group demonstrated that IL-6^−/−^ mice showed more oxidative stress with increased cox-2 expression and ERK phosphorylation, while superoxide dismutase activity was decreased [14]. More recently, the use of dimethylthiourea (DMTU), a hydroxyl radical scavenger, was shown to protect mice from cisplatin-induced AKI by increasing IL-6, Bcl-xL and Nrf2 [13]. Thus, these data indicate that IL-6 is involved in AKI protection by controlling cell death and through upregulation of anti-oxidative stress factors. Our results support this mechanism, as elevated IL-6 expression is observed in the kidneys of the exercised group (CIS-EX), as shown in Figure 4B. Cisplatin seems to induce IL-6 on its own, which may be acting as negative feedback for the inflammatory response, and exercised mice express even greater levels of IL-6 (Figure 4B), as if they were more prone to produce this cytokine. In the liver, IL-6 is responsible for liver regeneration, hepatoprotection, antiapoptosis and antinecrosis via STAT3 [34]. This cytokine is also produced by skeletal muscle fibers in response to physical exercise and associated with the anti-inflammatory response [35]. It has been reported that an increase in IL-6 levels depends on the intensity and duration of exercise [36] and is more pronounced in animals exposed to an overtraining protocol than to a moderate training protocol. Here, we did not notice any difference in IL-6 expression in muscle cells (Figure 4A) from exercised animals. This could be explained by the fact that samples were harvested three days after the cessation of training. According to Gholamnezhad *et al.,* a slight decrease in IL-6 is observed even 24 hours after exercise [37]. Therefore, it is very likely that local IL-6 production is responsible for the AKI protection.

We then decided to investigate Nrf2, the most potent inducer of antioxidant responsive element (ARE). This transcription factor is also able to induce IL-6 via ARE within its promoter [23]. In this study, we noticed no difference of Nrf2 level in the kidneys from exercised mice (Figure 5A). Nevertheless, another antioxidant enzyme that also contains ARE in its promoter, HO-1, was shown to be overexpressed in the kidneys of exercised mice compared to sedentary groups (Figures 5B, C and D). Several studies have demonstrated the importance of HO-1 in kidney protection. HO-1-deficient mice were shown to be more susceptible to renal injury caused by cisplatin [38], while metalloporphyrins and other inducers of HO-1 induced an anti-apoptotic response, protecting kidneys from cisplatin-induced injury [39]–[42]. Accordingly, our data suggest that exercise increases HO-1 expression in the kidney, possibly contributing to the protective phenotype.

## Conclusions

In summary, we showed that aerobic exercise was able to diminish cisplatin-induced AKI by promoting IL-6 and HO-1 expression in the kidney, which decreases inflammation and cell death in that organ. This study suggests that the introduction of physical exercise before and during chemotherapy could attenuate the side effects caused by the drug and improve quality of life for patients.

## References

[1] Pabla N, Dong Z (2008) Cisplatin nephrotoxicity: mechanisms and renoprotective strategies. Kidney Int 73: 994–1007. 18272962 10.1038/sj.ki.5002786

[2] Verstappen CC, Heimans JJ, Hoekman K, Postma TJ (2003) Neurotoxic complications of chemotherapy in patients with cancer: clinical signs and optimal management. Drugs 63: 1549–1563. 12887262 10.2165/00003495-200363150-00003

[3] Wensing KU, Ciarimboli G (2013) Saving ears and kidneys from cisplatin. Anticancer Res 33: 4183–4188. 24122981

[4] Garcia JM, Scherer T, Chen JA, Guillory B, Nassif A, et al (2013) Inhibition of cisplatin-induced lipid catabolism and weight loss by ghrelin in male mice. Endocrinology 154: 3118–3129. 23832960 10.1210/en.2013-1179 PMC3749475

[5] Arruda AP, Milanski M, Romanatto T, Solon C, Coope A, et al (2010) Hypothalamic actions of tumor necrosis factor alpha provide the thermogenic core for the wastage syndrome in cachexia. Endocrinology 151: 683–694. 19996183 10.1210/en.2009-0865

[6] Benedetti G, Fredriksson L, Herpers B, Meerman J, van de Water B, et al (2013) TNF-alpha-mediated NF-kappaB survival signaling impairment by cisplatin enhances JNK activation allowing synergistic apoptosis of renal proximal tubular cells. Biochem Pharmacol 85: 274–286. 23103562 10.1016/j.bcp.2012.10.012

[7] Yao X, Panichpisal K, Kurtzman N, Nugent K (2007) Cisplatin nephrotoxicity: a review. Am J Med Sci 334: 115–124. 17700201 10.1097/MAJ.0b013e31812dfe1e

[8] Zhang B, Ramesh G, Norbury CC, Reeves WB (2007) Cisplatin-induced nephrotoxicity is mediated by tumor necrosis factor-alpha produced by renal parenchymal cells. Kidney Int 72: 37–44. 17396112 10.1038/sj.ki.5002242

[9] Ramesh G, Brian Reeves W (2006) Cisplatin increases TNF-alpha mRNA stability in kidney proximal tubule cells. Ren Fail 28: 583–592. 17050242 10.1080/08860220600843839

[10] Tadagavadi RK, Reeves WB (2010) Endogenous IL-10 attenuates cisplatin nephrotoxicity: role of dendritic cells. J Immunol 185: 4904–4911. 20844196 10.4049/jimmunol.1000383 PMC3169908

[11] Deng J, Kohda Y, Chiao H, Wang Y, Hu X, et al (2001) Interleukin-10 inhibits ischemic and cisplatin-induced acute renal injury. Kidney Int 60: 2118–2128. 11737586 10.1046/j.1523-1755.2001.00043.x

[12] Hopps E, Canino B, Caimi G (2011) Effects of exercise on inflammation markers in type 2 diabetic subjects. Acta Diabetol 48: 183–189. 21431832 10.1007/s00592-011-0278-9

[13] Mitazaki S, Hashimoto M, Matsuhashi Y, Honma S, Suto M, et al (2013) Interleukin-6 modulates oxidative stress produced during the development of cisplatin nephrotoxicity. Life Sci 92: 694–700. 23384965 10.1016/j.lfs.2013.01.026

[14] Mitazaki S, Honma S, Suto M, Kato N, Hiraiwa K, et al (2011) Interleukin-6 plays a protective role in development of cisplatin-induced acute renal failure through upregulation of anti-oxidative stress factors. Life Sci 88: 1142–1148. 21570986 10.1016/j.lfs.2011.04.016

[15] Mitazaki S, Kato N, Suto M, Hiraiwa K, Abe S (2009) Interleukin-6 deficiency accelerates cisplatin-induced acute renal failure but not systemic injury. Toxicology 265: 115–121. 19833167 10.1016/j.tox.2009.10.005

[16] Correa-Costa M, Amano MT, Camara NO (2012) Cytoprotection behind heme oxygenase-1 in renal diseases. World J Nephrol 1: 4–11. 24175236 10.5527/wjn.v1.i1.4 PMC3782207

[17] Schwartz AL, Mori M, Gao R, Nail LM, King ME (2001) Exercise reduces daily fatigue in women with breast cancer receiving chemotherapy. Med Sci Sports Exerc 33: 718–723. 11323538 10.1097/00005768-200105000-00006

[18] Jensen W, Baumann FT, Stein A, Bloch W, Bokemeyer C, et al. (2014) Exercise training in patients with advanced gastrointestinal cancer undergoing palliative chemotherapy: a pilot study. Support Care Cancer. 10.1007/s00520-014-2139-x 24531742

[19] Eickmeyer SM, Gamble GL, Shahpar S, Do KD (2012) The role and efficacy of exercise in persons with cancer. PM R 4: 874–881. 23174553 10.1016/j.pmrj.2012.09.588

[20] Wang S, Wei Q, Dong G, Dong Z (2013) ERK-mediated suppression of cilia in cisplatin-induced tubular cell apoptosis and acute kidney injury. Biochim Biophys Acta 1832: 1582–1590. 23727409 10.1016/j.bbadis.2013.05.023 PMC3752396

[21] Bacurau RF, Belmonte MA, Seelaender MC, Costa Rosa LF (2000) Effect of a moderate intensity exercise training protocol on the metabolism of macrophages and lymphocytes of tumour-bearing rats. Cell Biochem Funct 18: 249–258. 11180287 10.1002/1099-0844(200012)18:4<249::AID-CBF879>3.0.CO;2-2

[22] Roudier E, Forn P, Perry ME, Birot O (2012) Murine double minute-2 expression is required for capillary maintenance and exercise-induced angiogenesis in skeletal muscle. FASEB J 26: 4530–4539. 22835827 10.1096/fj.12-212720 PMC3475253

[23] Wruck CJ, Streetz K, Pavic G, Gotz ME, Tohidnezhad M, et al (2011) Nrf2 induces interleukin-6 (IL-6) expression via an antioxidant response element within the IL-6 promoter. J Biol Chem 286: 4493–4499. 21127061 10.1074/jbc.M110.162008 PMC3039356

[24] Li N, Alam J, Venkatesan MI, Eiguren-Fernandez A, Schmitz D, et al (2004) Nrf2 is a key transcription factor that regulates antioxidant defense in macrophages and epithelial cells: protecting against the proinflammatory and oxidizing effects of diesel exhaust chemicals. J Immunol 173: 3467–3481. 15322212 10.4049/jimmunol.173.5.3467

[25] Choi AM, Alam J (1996) Heme oxygenase-1: function, regulation, and implication of a novel stress-inducible protein in oxidant-induced lung injury. Am J Respir Cell Mol Biol 15: 9–19. 8679227 10.1165/ajrcmb.15.1.8679227

[26] Miller RP, Tadagavadi RK, Ramesh G, Reeves WB (2010) Mechanisms of Cisplatin nephrotoxicity. Toxins (Basel) 2: 2490–2518. 22069563 10.3390/toxins2112490 PMC3153174

[27] Pereira WO, Amarante-Mendes GP (2011) Apoptosis: a programme of cell death or cell disposal? Scand J Immunol 73: 401–407. 21223349 10.1111/j.1365-3083.2011.02513.x

[28] Ramesh G, Reeves WB (2002) TNF-alpha mediates chemokine and cytokine expression and renal injury in cisplatin nephrotoxicity. J Clin Invest 110: 835–842. 12235115 10.1172/JCI15606 PMC151130

[29] Liu M, Chien CC, Burne-Taney M, Molls RR, Racusen LC, et al (2006) A pathophysiologic role for T lymphocytes in murine acute cisplatin nephrotoxicity. J Am Soc Nephrol 17: 765–774. 16481417 10.1681/ASN.2005010102

[30] Jenkins NT, Padilla J, Arce-Esquivel AA, Bayless DS, Martin JS, et al (2012) Effects of endurance exercise training, metformin, and their combination on adipose tissue leptin and IL-10 secretion in OLETF rats. J Appl Physiol (1985) 113: 1873–1883. 23019312 10.1152/japplphysiol.00936.2012 PMC3544496

[31] Lira FS, Rosa JC, Zanchi NE, Yamashita AS, Lopes RD, et al (2009) Regulation of inflammation in the adipose tissue in cancer cachexia: effect of exercise. Cell Biochem Funct 27: 71–75. 19226603 10.1002/cbf.1540

[32] Kishimoto T (2010) IL-6: from its discovery to clinical applications. Int Immunol 22: 347–352. 20410258 10.1093/intimm/dxq030

[33] Bettelli E, Carrier Y, Gao W, Korn T, Strom TB, et al (2006) Reciprocal developmental pathways for the generation of pathogenic effector TH17 and regulatory T cells. Nature 441: 235–238. 16648838 10.1038/nature04753

[34] Taub R (2003) Hepatoprotection via the IL-6/Stat3 pathway. J Clin Invest 112: 978–980. 14523032 10.1172/JCI19974 PMC198534

[35] Pedersen BK, Steensberg A, Schjerling P (2001) Muscle-derived interleukin-6: possible biological effects. J Physiol 536: 329–337. 11600669 10.1111/j.1469-7793.2001.0329c.xd PMC2278876

[36] Petersen AM, Pedersen BK (2006) The role of IL-6 in mediating the anti-inflammatory effects of exercise. J Physiol Pharmacol 57 Suppl 1043–51. 17242490

[37] Gholamnezhad Z, Boskabady MH, Hosseini M, Sankian M, Khajavi Rad A (2014) Evaluation of immune response after moderate and overtraining exercise in wistar rat. Iran J Basic Med Sci 17: 1–8. 24592300 PMC3938879

[38] Shiraishi F, Curtis LM, Truong L, Poss K, Visner GA, et al (2000) Heme oxygenase-1 gene ablation or expression modulates cisplatin-induced renal tubular apoptosis. Am J Physiol Renal Physiol 278: F726–736. 10807584 10.1152/ajprenal.2000.278.5.F726

[39] Pan H, Shen K, Wang X, Meng H, Wang C, et al (2014) Protective effect of metalloporphyrins against cisplatin-induced kidney injury in mice. PLoS One 9: e86057. 24454954 10.1371/journal.pone.0086057 PMC3891880

[40] Tayem Y, Green CJ, Motterlini R, Foresti R (2014) Isothiocyanate-cysteine conjugates protect renal tissue against cisplatin-induced apoptosis via induction of heme oxygenase-1. Pharmacol Res 81C: 1–9. 10.1016/j.phrs.2014.01.001 24434421

[41] Sahin K, Orhan C, Tuzcu M, Muqbil I, Sahin N, et al (2014) Comparative in vivo evaluations of curcumin and its analog difluorinated curcumin against Cisplatin-induced nephrotoxicity. Biol Trace Elem Res 157: 156–163. 24415068 10.1007/s12011-014-9886-x

[42] Sahin K, Tuzcu M, Sahin N, Ali S, Kucuk O (2010) Nrf2/HO-1 signaling pathway may be the prime target for chemoprevention of cisplatin-induced nephrotoxicity by lycopene. Food Chem Toxicol 48: 2670–2674. 20603177 10.1016/j.fct.2010.06.038

